# Data of a high temperature heat injection test

**DOI:** 10.1016/j.dib.2021.107035

**Published:** 2021-04-03

**Authors:** Stefan Heldt, Bo Wang, Linwei Hu, Götz Hornbruch, Klas Lüders, Ulrike Werban, Sebastian Bauer

**Affiliations:** a Institute of Geosciences, Christian-Albrechts-University Kiel, Kiel, Germany; b Dep. of Monitoring and Exploration Technologies, Helmholtz Centre for Environmental Research – UFZ, Leipzig, Germany

**Keywords:** High temperature heat injection test, Buoyancy flow, Long-term temperature monitoring, Multi-level pumping test

## Abstract

This document compiles the data related to a high temperature heat injection test, which was carried out at an injection temperature of 74 °C in a shallow aquifer and is presented by Heldt et al. [1]. The data set contains transient measurements of temperatures at 18 wells in 10 depths and measurements of the experimental boundary conditions (injection temperature and flow rate) at a temporal resolution of up to 1 min. The spatial configuration and the technical details about where and how the data have been measured are provided. In addition, data of a multilevel multi well pumping test are shown. The presented data is useful to gain insights into the thermohydraulic processes induced by a high temperature heat injection test and can furthermore be used for the development and verification of numerical models of the presented experiment and similar applications like high temperature aquifer thermal energy storage.

## Specifications Table

**Table:** 

Subject	Energy, Sustainability and the Environment
Specific subject area	Subsurface thermo-hydraulic processes induced by a high temperature heat injection test
Type of data	Table Graph Figure
How data were acquired	Temperature measurements: T-type thermocouples Channel Expansion Modules: dataTaker CEM20 Temperature logging: dataTaker DT85 and dataTaker dEX 1.84.030 software Injection temperature: Easytemp TMR31 Injection flow rate: Proline Promag P 300 Pumping test pressure logging: Solinst Levelogger 3001 LT M20 (observation wells), Solinst Levelogger 3001 LT M30 (pumping well) and Solinst Barologger 3001 (atmospheric pressure)
Data format	Raw and processed
Parameters for data collection	Subsurface temperature data was recorded in °C. Injection temperature was recorded in °C and injection flow rate was recorded in l/min. Pressure was recorded in kPa, m and cm (water column). Coordinates were recorded in UTM (WGS84; zone 33 U) format.
Description of data collection	Subsurface temperature was measured by T-type thermocouples and the data was logged in different intervals varying from 1 – 20 min using a dataTaker DT85 data logging unit. The number of input ports of the data taker was increased by using dataTaker CEM20 Channel Expansion Modules. Injection temperature and injection flow rate were recorded at an interval of 1 min. Pressure was recorded at an interval of 1 s or 2 s.
Data source location	TestUM-Aquifer test site near Wittstock/Dosse, Brandenburg, Germany 53.194349 N, 12.504932 E (Injection Well)
Data accessibility	Repository name: Mendeley Data Data identification number: http://dx.doi.org/10.17632/rjzt4hjb8h.1 Direct URL to data: http://dx.doi.org/10.17632/rjzt4hjb8h.1
Related research article	S. Heldt, B. Wang, L. Hu, G. Hornbruch, K. Lüders, U. Werban, S. Bauer: Numerical Investigation of a High Temperature Heat Injection Test, J. Hydrol., 2021. https://doi.org/10.1016/j.jhydrol.2021.126229

## Value of the Data

• The presented experimental data is useful to gain insights into the thermohydraulic processes induced by a high temperature heat injection in a shallow aquifer and the related parameter sensitivities.
• The data is interesting for the scientific audience working on thermal tracer tests, aquifer thermal energy storage and buoyancy driven flow.
• The data can be used for the development and verification of numerical models of the presented heat injection test and similar applications like high temperature aquifer thermal energy storage.

## 1 Data Description

**Fig. 1:**
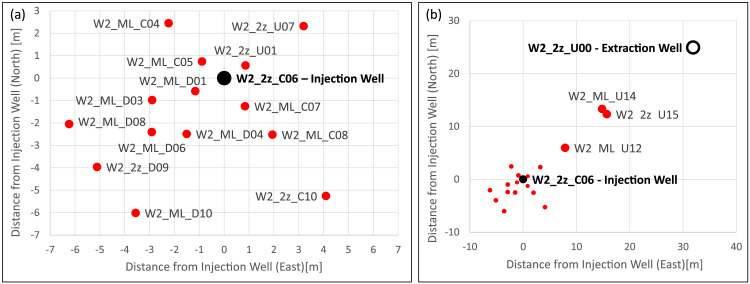
Field test site with (a) the monitoring wells near the injection well (W2_2z_C06) and (b) the wells of the whole test field including the extraction well (W2_2z_U00).

Fig. 1 shows the positions of the wells at the test site for temperature monitoring, which include an injection well (W2_2z_C06), an extraction well (W2_2z_U00) and 17 monitoring wells. Table 1 presents the relative coordinates of these wells’ positions and additionally includes the well NMR04, which is not used for temperature monitoring, but instead for the multilevel pumping test. The corresponding raw data, i.e. the coordinates in UTM format, are accessible in the data repository.

**Table 1:** Distances of the wells to the injection well in east and north.

Well	Distance E [m]	Distance N [m]
W2_2z_C06	0.00	0.00
W2_2z_C10	4.10	−5.26
W2_2z_D09	−5.11	−3.96
W2_2z_U00	31.90	24.93
W2_2z_U01	0.86	0.56
W2_2z_U07	3.20	2.32
W2_2z_U15	15.70	12.32
W2_ML_C04	−2.23	2.45
W2_ML_C05	−0.89	0.74
W2_ML_C07	0.84	−1.26
W2_ML_C08	1.94	−2.52
W2_ML_D01	−1.16	−0.58
W2_ML_D03	−2.90	−0.98
W2_ML_D04	−1.50	−2.49
W2_ML_D06	−2.91	−2.41
W2_ML_D08	−6.23	−2.05
W2_ML_D10	−3.31	−5.53
W2_ML_U12	7.88	5.95
W2_ML_U14	14.81	13.31
NMR04	−3.97	0.39

**Fig. 2:**
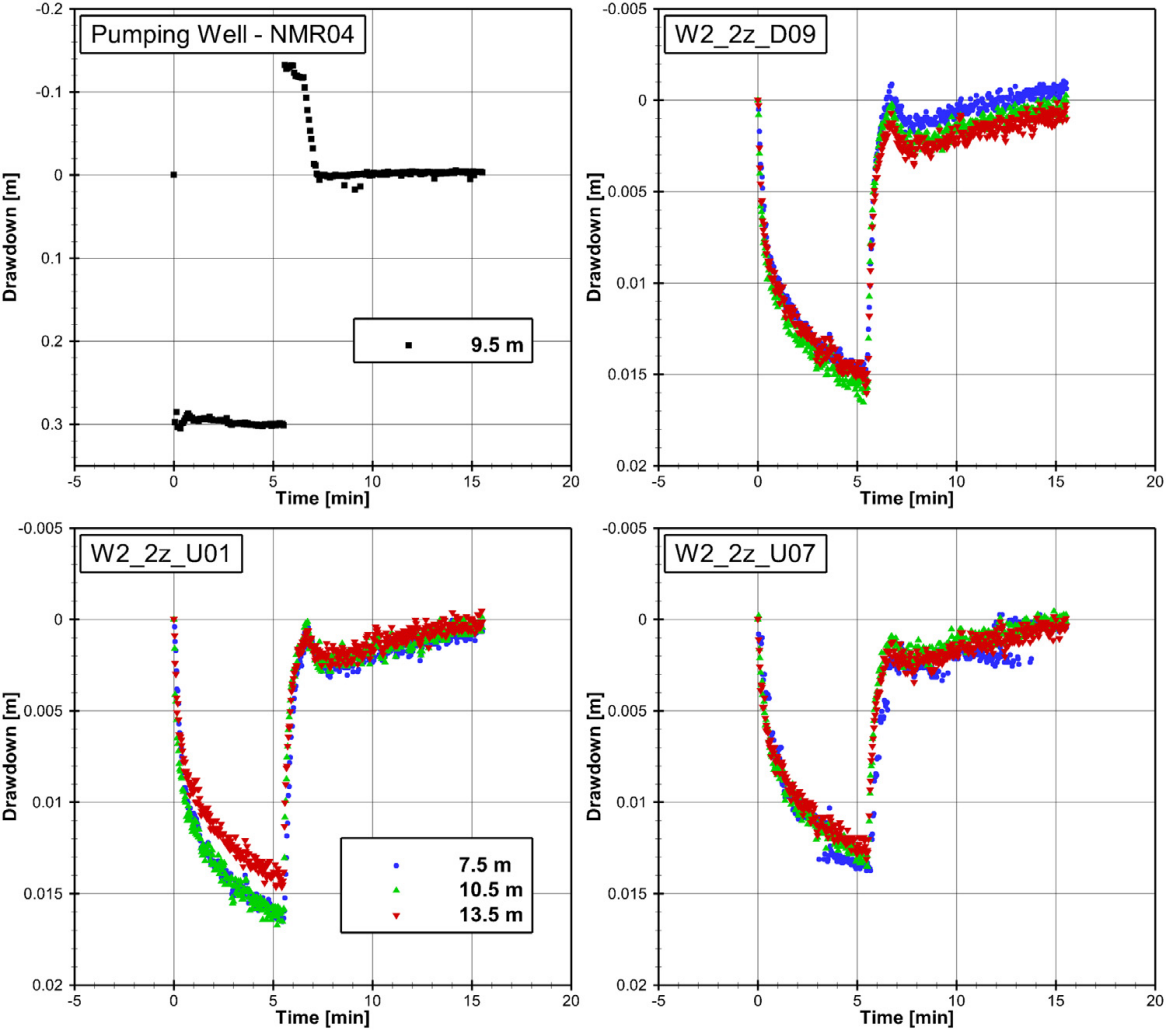
Drawdown curves at the pumping well and three observation wells in three different depths for the pumping test conducted at well NMR04 in 9,10 m below ground limit. The depth given in the legend stands for the centre of the well screen, which is 1 m long.

Fig. 2 shows the drawdown curves of the pumping test conducted at the well NMR04 in 9 to 10 m below ground level (b.g.l.). The drawdown at the pumping well reaches a maximum of about 0.3 m, while a maximum drawdown of about 0.015 m is reached at the observation wells. The three curves from the three screens of the observation wells show slightly different drawdowns. The smallest drawdown at the wells W2_2z_U01 and W2_2z_U07 is observed at a depth of 13.5 m, while at W2_2z_D09 the drawdown in 7.5 m and 13.5 m are approximately the same. A water level increase of 0.13 m over the initial level can be observed at the pumping well when pumping stopped after about 5.5 min. This effect is caused by backflow of pipe water into the well. A corresponding effect with lower magnitude is shown at the observation wells.

The measured raw data of ten drawdown curves as well as the processed data are provided in the data repository referenced above with the time since pumping started in seconds and the drawdown in meters. The naming of the processed data files (e.g., “NMR4_W2_2z_D09_S1_R2_pump_recovery_20,190,325″) includes the pumping well (NMR4), the observation well (W2_2z_D09), the pumped screen section (S1), the observation screen section (R2) and the date. The processed data is corrected for the influence of atmospheric pressure fluctuations.

**Fig. 3:**
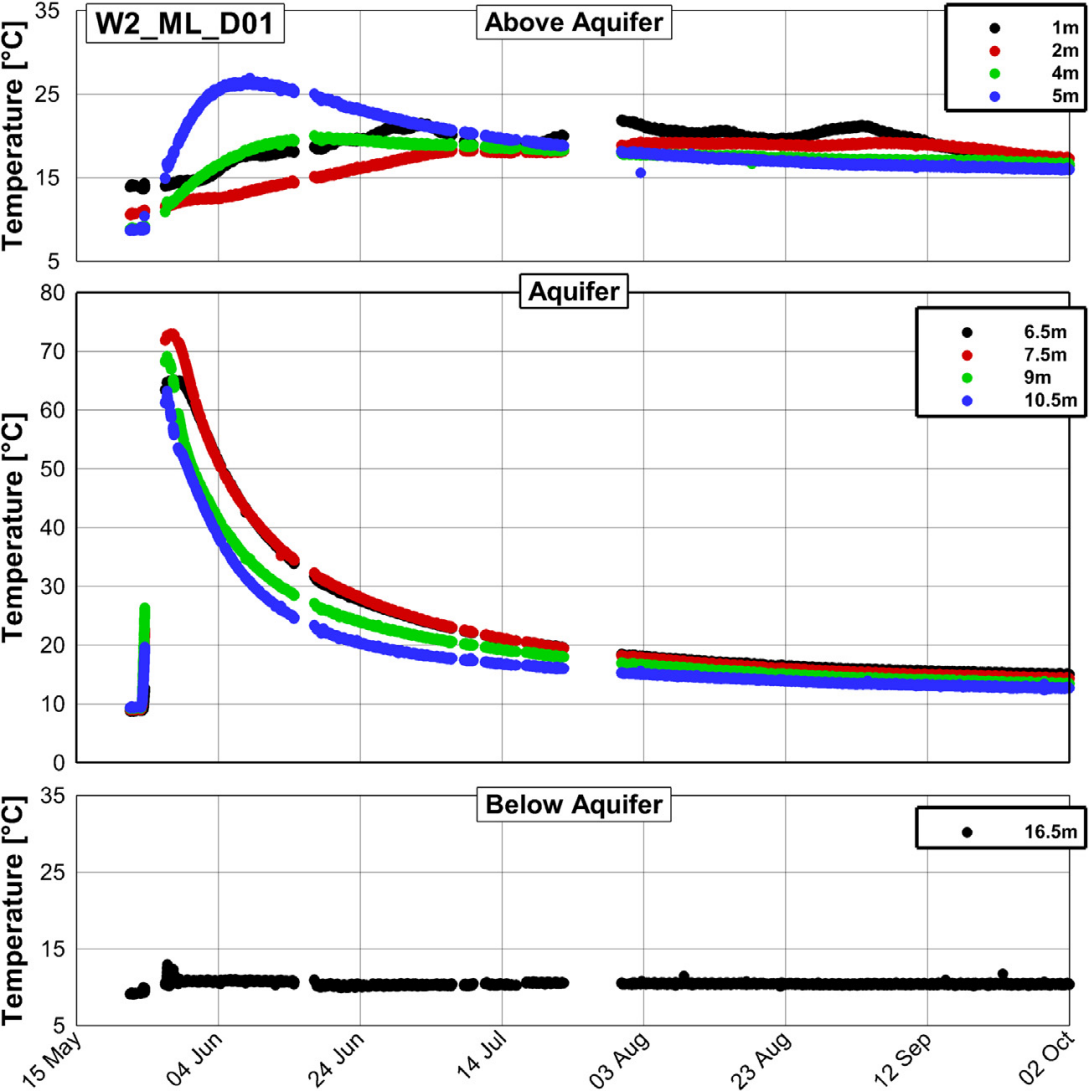
Temperature time series at the well W2_ML_D01, which is situated approximately 1.2 m downstream from the injection well (compare Fig. 1).

**Fig. 4:**
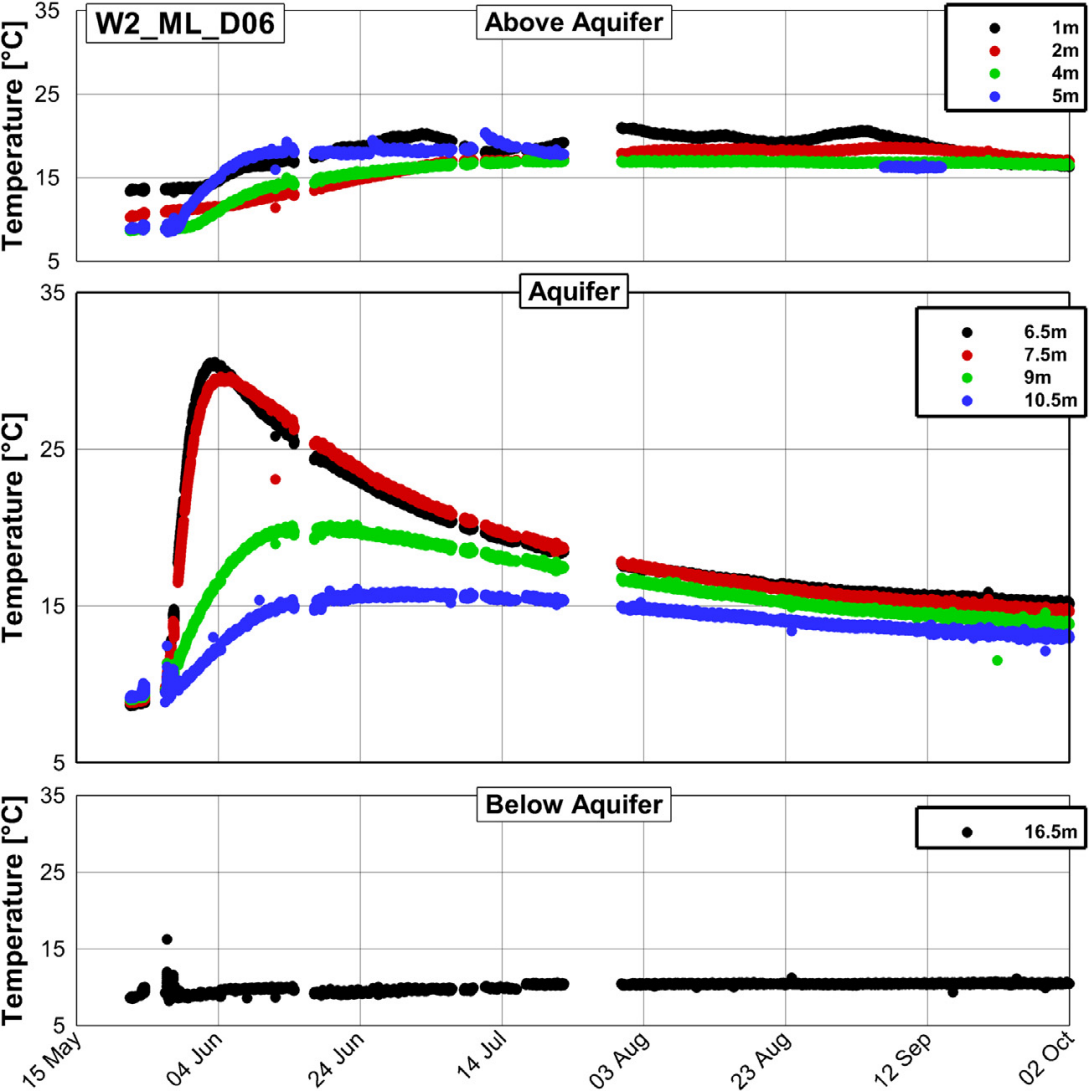
Temperature time series at the well W2_ML_D06, which is situated approximately 3.1 m downstream from the injection well (compare Fig. 1).

**Fig. 5:**
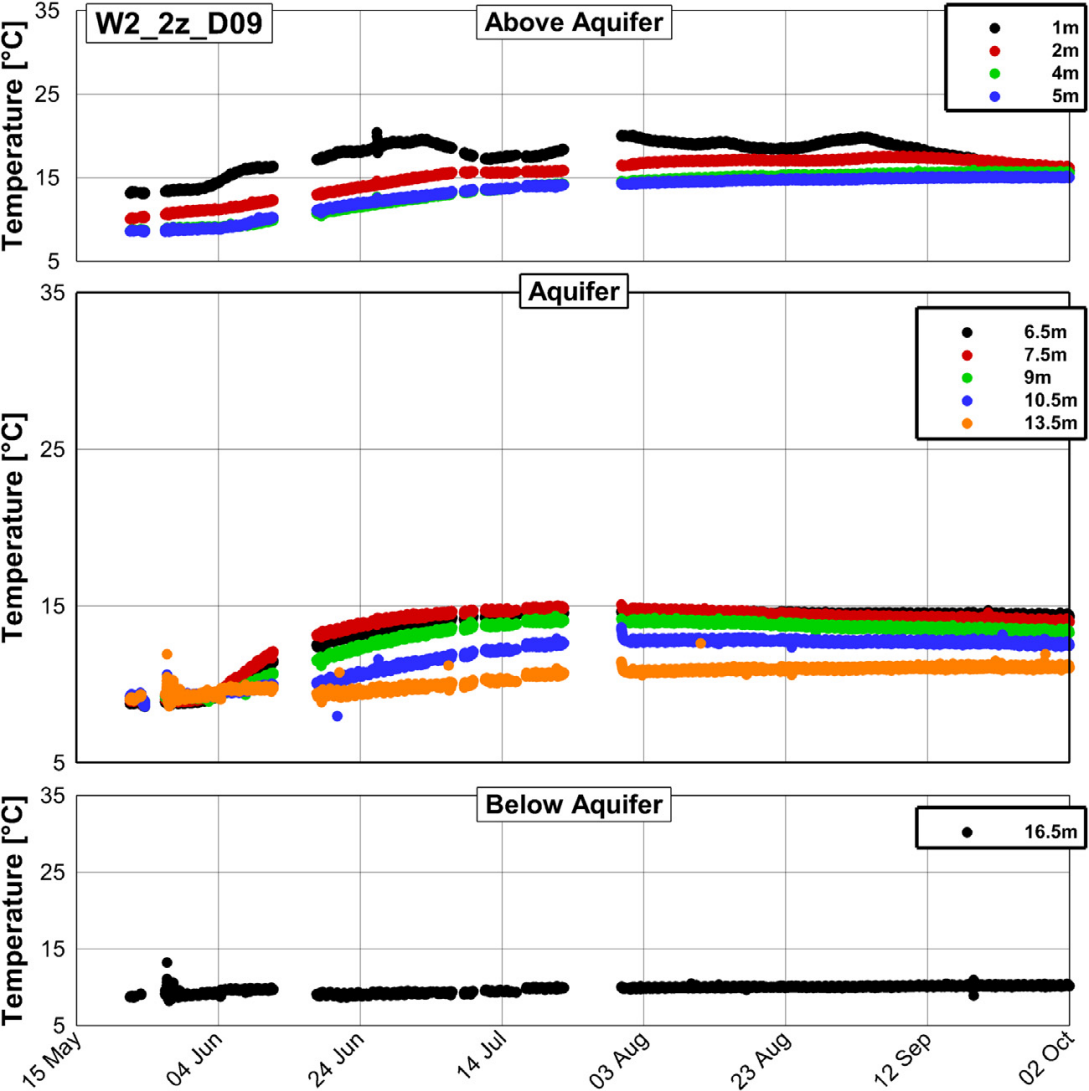
Temperature time series at the well W2_2z_D09, which is situated approximately 6.5 m downstream from the injection well (compare Fig. 1).

Figs. 3, 4 and 5 present examples of measured temperature series at different depths at the wells W2_ML_D01, W2_ML_D06 and W2_2z_D09, respectively.

All three figures display a rise of temperature in the aquifer relative to the initial temperature of approximately 9 °C. The temperature peak is most pronounced at W2_ML_D01 with a maximum temperature of 73 °C, while at W2_ML_D06 and W2_2z_D09 maximum temperatures of 31 and 15 °C were found, respectively. The measured temperature series of the 17 observation wells and the injection well as exemplarily shown in Figs. 3, 4 and 5 are summarized in the data repository in a Microsoft Excel spreadsheet (Temperature_time_series/Processed/Temperature_time_series_processed.xlsx).

The injection temperature and flow rate are shown by [1]. Processed and raw time series of the injection temperature and flow rate are provided in the data repository at an interval of 1 min. Injection temperature data between 03:00 h to 03:15 h each day is omitted due to distortions by geoelectrical measurements.

## 2 Experimental Design, Materials and Methods

The data compiled in this document was collected during a high temperature heat injection test in a shallow aquifer and the preceding field site investigation described in [1]. The field site is located approximately 100 km north of Berlin near the town of Wittstock/Dosse, Brandenburg in northern Germany. Four geological layers were identified: the unsaturated zone (ground level to 3 m b.g.l), the upper aquitard (3 to 6 m b.g.l), the aquifer (6 to 15 m b.g.l.) and the lower aquitard (15 to 20 m b.g.l.). The groundwater flow direction is approximately from north-east to south-west.

Water was pumped from the aquifer through the extraction well during the heat injection test, heated to a temperature of about 74 °C and injected into the aquifer at the injection well. Both the flow rate and the temperature of the injected water (see [1] or uploaded data) were measured continuously at the wellhead of the injection well. The injection flow rate was measured by a Proline Promag P 300 flowmeter (accuracy of 0.1 l/min; Endress+Hauser GmbH+Co. KG, Weil am Rhein, Germany) and the injection temperature by an Easytemp TMR31 sensor (accuracy of 0.4 °C; Endress+Hauser GmbH+Co. KG, Weil am Rhein, Germany).

The temperatures at the injection well and the monitoring wells were measured by thermocouple sensors (Type T by Labfacility Ltd, Bognor Regis, United Kingdom and Type T, Class 1 by OMEGA Engineering GmbH, Deckenpfronn, Germany; with a resolution of 1 °C) in ten depths: in 1 m, 2 m, 4 m, 5 m, 6.5 m, 7.5 m, 9 m, 10.5 m, 13.5 m and 16.5 m b.g.l., resulting in a total number of 180 sensors. The sensors were attached to the outer side of the tubing and connected to a dataTaker DT85 data logging unit (Thermo Fisher Scientific Pty Ltd, Waltham, USA) via five dataTaker Channel Expansion Modules 20 (CEMs; Thermo Fisher Scientific Pty Ltd, Waltham, USA). The CEMs were installed at 0.5 m b.g.l. to minimize the influence of fluctuating surface temperatures on thermocouple measurements. The cables connecting the CEMs to the data logging unit and the thermocouples were installed at 0.3 m b.g.l. The DT85 data taker was accessed via the software dEX 1.84.030 (Thermo Fisher Scientific Pty Ltd, Waltham, USA). The measuring interval of the subsurface temperature measurements was successively increased from 1 min at the start of the experiment in May 2019 to 20 min from July 2019 onwards.

No data has been recorded from 23rd May 9:20 am to 24th May 0:08 am due to technical problems related to the logging unit. Data gaps furthermore occurred from 24th May 3:47 pm to 27th May 12:23 pm and at several other times. A complete list of data gaps is in the data repository. Furthermore, several thermocouples failed either from the start of the experiment or from a later time during the experiment. The sensors in 13.5 m depth at the wells W2_ML_D01 and W2_ML_D06 in Figs. 3 and 4, respectively, failed from the start and the sensor in 5 m depth at W2_ML_D06 in Fig. 4 failed from 22rd July 6:20 pm. The sensor in 5 m depth at W2_ML_D06 seems to work again between 5th September 9.20 pm and 13th September 11:40 pm, but fails again afterwards. In total, 11 of the 180 thermocouples installed failed until November 2019. A list of the failed thermocouples is provided in the data repository.

The available data set of measured temperature has been processed using a self-written program (PostProcessing.exe), which is provided in the data repository (Temperature_time_series\Processing_scripts\DataProcessing_executable) together with a documentation on how to use it. This program is used for screening the raw data, organizing it and writing it out as the format which can be visualized by the Program Tecplot 360 Ex. In the processed data, some outliers were only removed when several consecutive erroneous measurements occurred and consequently some single outliers can still be found (see Figs. 3 to 5). A python script (Temperature_time_series\Processing_scripts\Python_script\2_Write_Smoothed_Data.py in the data repository) was used to write the output from the self-written program to a Microsoft Excel spreadsheet.

The pumping test presented in Fig. 2 was carried out at the well NMR04 in 9 to 10 m b.g.l. and the three drawdown curves of each observation well were measured at the three well screens R1 (7 to 8 m b.g.l.), R2 (10 to 11 m b.g.l.) and R3 (13 to 14 m b.g.l.). The pumping rate was manually logged as 5.82 l/min during the 333 s of groundwater abstraction. The water pressure was logged by a Solinst Levelogger 3001 LT M30 pressure sensor (accuracy: ±1.5 cm; Solinst Canada Ltd, Georgetown, Canada) in the pumping well and by several Solinst Levelogger 3001 LT M20 pressure sensors (accuracy: ±1 cm) in the observation wells. An additional Solinst Barologger 3001 (accuracy: ± 0.05 kPa) was added at one observation well to measure the changes on the atmospheric pressure.

## CRediT Author Statement

**Stefan Heldt:** Data curation, Formal analysis, Investigation, Software, Visualization, Writing - original draft, Writing - review & editing; **Bo Wang:** Software, Writing - original draft; **Linwei Hu:** Data curation, Formal analysis, Investigation; **Götz Hornbruch:** Conceptualization, Data curation, Investigation, Funding acquisition, Project administration; **Klas Lüders:** Data curation, Investigation; **Ulrike Werban:** Data curation, Investigation; **Sebastian Bauer:** Conceptualization, Funding acquisition, Writing - original draft.

## Declaration of Competing Interest

The authors declare that they have no known competing financial interests or personal relationships which have, or could be perceived to have, influenced the work reported in this article.
